# Identification of a mutated BHK-21 cell line that became less susceptible to Japanese encephalitis virus infection

**DOI:** 10.1186/1743-422X-8-115

**Published:** 2011-03-14

**Authors:** Tianbing Ding, Wei Zhang, Wenyu Ma, Junping Ren

**Affiliations:** 1 Department of Microbiology, the Fourth Military Medical University, Xi'an, Shaanxi, 710032, PR China; 2 Department of Biopharmaceutics, School of Pharmacy, the Fourth Military Medical University, Xi'an, Shaanxi, 710032, PR China

## Abstract

The pathogenesis of Japanese encephalitis virus (JEV) is not definitely elucidated as the initial interaction between virus and host cell receptors required for JEV infection is not clearly defined yet. Here, in order to discover those membrane proteins that may be involved in JEV attachment to or entry into virus permissive BHK-21 cells, a chemically mutated cell line (designated 3A10-3F) that became less susceptible to JEV infection was preliminarily established and selected by repeated low moi JEV challenges and RT-PCR detection for viral RNA *E *gene fragment. The susceptibility to JEV of 3A10-3F cells was significantly weakened compared with parental BHK-21 cells, verified by indirect immunofluorescence assay, virus plague formation assay, and flow cytometry. Finally, two-dimensional electrophoresis (2-DE) coupled with LC-MS/MS was utilized to recognize the most differentially expressed proteins from membrane protein extracts of 3A10-3F and BHK-21 cells respectively. The noted discrepancy of membrane proteins included calcium binding proteins (annexin A1, annexin A2), and voltage-dependent anion channels proteins (VDAC 1, VDAC 2), suggesting that these molecules may affect JEV attachment to and/or entry into BHK-21 cells and worthy of further investigation.

## Findings

Japanese encephalitis virus (JEV), a member of genus *Flavivirus *in the family *Flaviviridae*, is the causative agent of Japanese encephalitis (JE), the mosquito-borne viral encephalitis epidemic in eastern, southeastern and southern Asia, leading to an estimated ~50,000 infections annually, of which ~15,000 will die and up to 50% of survivors are left with severe residual neurological complications [1,2]. During the past decades, JE is spreading beyond its traditional boundaries and has reported from previously unaffected areas such as Saipan islands, Pakistan and northern Australia [3-5]. Coupled with a high rate of mortality and residual neurological complications in survivors, it makes JE a serious public health problem in tropical and subtropical areas in the world.

The first step of virus infection requires the interaction between virus attachment proteins (VAPs) and cellular receptors, which is known to contribute to host range, tissue tropism and viral pathogenesis. In the cases of flaviviruses including JEV, envelope glycoprotein E, protruding as spikes on the surface of virions, is considered to be the dominant antigen in mediating receptor binding and membrane fusion, hemagglutination, neutralization and virulence [6-8]. As of enzootic nature, JEV maintains a natural cycle among birds, pigs, and other vertebrate hosts by mosquitoes without serious sickness, and thus the cells from above species, such as African green monkey kidney cells (Vero), baby hamster kidney cells (BHK-21), and *Aedes albopictus *cells (C6/36) [9], are frequently applied in studies associated with JEV pathogenicity due to their ability to permit JEV entry and replication within them. Such broad tropism of JEV rationally suggests that most possibly there exists more than one cellular receptor responsible for virus binding and entry into susceptible cells above mentioned.

To date, little is known about JEV cellular receptors. Much earlier, a report stated that a 74 kDa protein on Vero cells was found to be capable of binding JEV and might be involved in virus uptake process [10]. A recent paper also indicated that several proteins on the surface of C6/36 cells with masses ranging from 35-80 kDa and 150-200 kDa may bind to JEV, but failed to identify specific proteins by mass spectroscopic fingerprint analysis [11]. In 2009, a paper reported that heat shock protein 70 is a putative receptor for JEV on mouse neuroblastoma (Neuro2a) cells [12]. Hence, the detailed interaction between JEV and its putative receptor(s) is not exclusively defined yet.

One of the most convincing methods to verify a putative virus receptor is to transfer the receptor gene into a cell line that cannot bind virus and later demonstrate that the receptor-negative cell acquires the ability to bind virus and permit virus to replicate within it after the receptor gene is regained [13]. For this reason, the availability of a specific virus receptor-negative/-defective cell line is usually a prerequisite for virus receptor confirmation. Unfortunately, such JEV receptor-negative/-defective cell line is not currently available after our extensive searching for animal and human cell lines, and thus it has to be established artificially.

**Figure 1:**
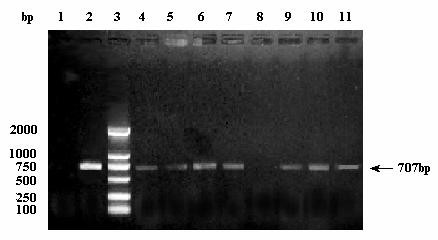
**Selection of mutated cell lines negative of JEV E gene RNA fragments by RT-PCR**. Eight single-cell clones of mutated cells (lanes 4-11) were detected in triplicate. The arrow indicates 707 bp fragments containing the conserved JEV E gene sequence. Only one single-cell clone, designated 3A10-3F, showed JEV RNA negative (lane 8). BHK-21 cells (lane 1) and BHK-21 cells infected with JEV (lane 2) were negative and positive controls respectively. Lane 3 was DL-2000 DNA marker.

**Figure 2:**
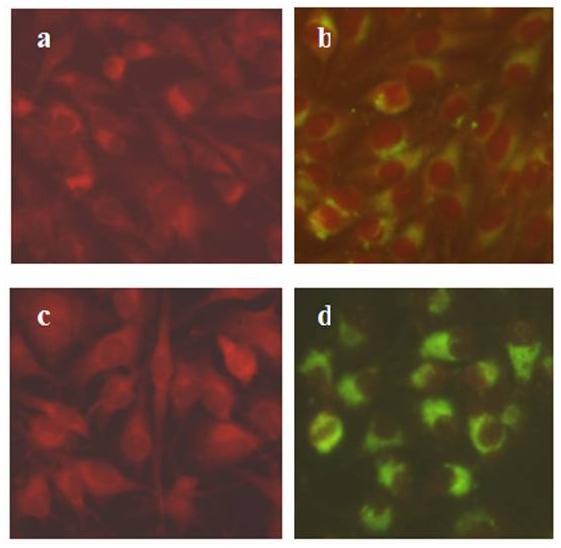
**JEV binding to 3A10-3F and BHK-21 cells measured by indirect immunofluorescence assay (IFA)**. JEV antigen were detected with anti-JEV mAb and FITC-conjugated goat anti-mouse IgG at 24 hr post-infection, and uninfected 3A10-3F cells (a) and BHK-21 cells (c) were negative controls. The cytoplasm and nuclei were stained red by Evans blue, and green fluorescent signals of viral antigen were significantly weaker in 3A10-3F cells (b) than in BHK-21 cells (d) (Magnification: ×400).

The successful identification of the cellular receptor for anthrax toxin provided us a practical strategy to create any specific virus receptor-negative/-defective cells [14]. Here, BHK-21 cells, permissive to JEV entry and replication, were subject to co-culture with a DNA alkylating mutagen ICR-191 to introduce random small DNA deletions and frame shift mutations in the genes of normally cultured BHK-21 cells under conditions that led to ~90% cell death. The survived cells were grown up but underwent a slow die-off for 4 weeks. These viable but mutated BHK-21 cells were successively subject to several rounds of infectious JEV challenge at a low moi of 0.1, subcloned by limiting dilution, and then each individual subclones was detected by RT-PCR for negative JEV *E *gene RNA fragment. RT-PCR primers were synthesized according to the RNA sequence of JEV SA-14 strain [15] from 1528 to 2234 of *E *gene conserved fragment, which were sense-strand primer, 5'-CGGAATTCGAGAAGTCACACTGGACTGTGAGCC-3', and antisense-strand primer, 5'-CGCTGCCAGTCTTTGAGCTCCCTTCAAAGT-3'. Finally, such a JEV RNA-negative mutated cell line, designated 3A10-3F, was picked out after two cycles of limiting dilution cloning from eight subclones of mutated BHK-21 cells (Figure 1). Further, the susceptibility of 3A10-3F to JEV infection was tested by indirect immunofluorescence assay (IFA), viral plaque formation assay, and flow cytometry. In IFA, JEV E protein synthesized within the cells was detected by monoclonal antibody against E protein. The results clearly displayed that fluorescent signals from 3A10-3F were much weaker (Figure 2b) than BHK-21 cells (Figure 2d) with the same dosage of JEV, while both negative controls of the two cell lines appeared the same (Figure 2a and Figure 2c).

**Figure 3:**
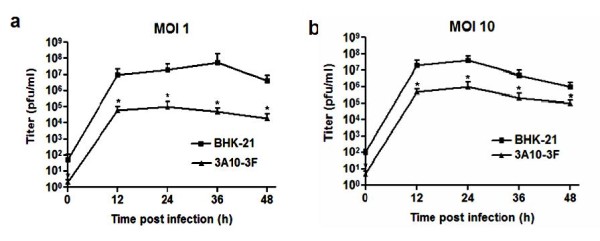
**JEV replication in 3A10-3F (black trianle) and BHK-21 (black square) cells measured by plaque formation assay**. Cells were infected with JEV at moi of 1 (a) and 10 (b). The viral titers were determined by plaque formation assay for culture supernatant samples harvested at 0, 12, 24, 36, and 48 hr post-infection. The numbers of virions detected in 3A10-3F cells was greatly reduced. The results displayed were the means of independent experiments performed in duplicate.

To reckon the susceptibility of 3A10-3F to JEV, virus plaque formation assay and flow cytometry were applied. It appeared that JEV was able to replicate in the same kinetic mode in either 3A10-3F or BHK-21 cells at 0-48 h post-infection (Figure 3), but the numbers of infectious virions detected by plaque formation assay in 3A10-3F cells were remarkably declined. At moi of 1, the highest JEV titer at 48 h post-infection from 3A10-3F cells was decreased by 2 orders of logarithm compared with that of titer from BHK-21 cells (Figure 3a). At moi of 10, the highest titer from 3A10-3F cells was lowered by 1 order of logarithm compared with that of from BHK-21 cells. Conspicuously there was a similar decline mode of virus replication in 3A10-3F cells compared with its parental cells between the two moi of JEV.

**Figure 4:**
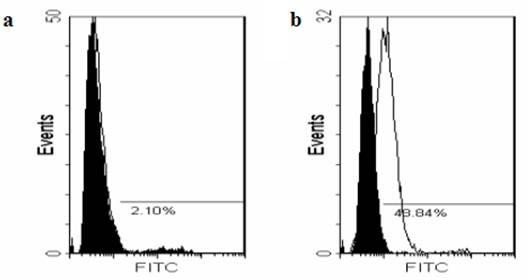
**JEV binding to 3A10-3F and BHK-21 cells measured by flow cytometry**. Cells were incubated with JEV for 1 hr and were stained with rabbit anti-JEV antibodies followed by FITC-conjugated goat anti-rabbit IgG. Limited binding of JEV to 3A10-3F cells was shown (2.10%, a), whereas JEV significantly bound BHK-21 cells (48.84%, b).

**Figure 5:**
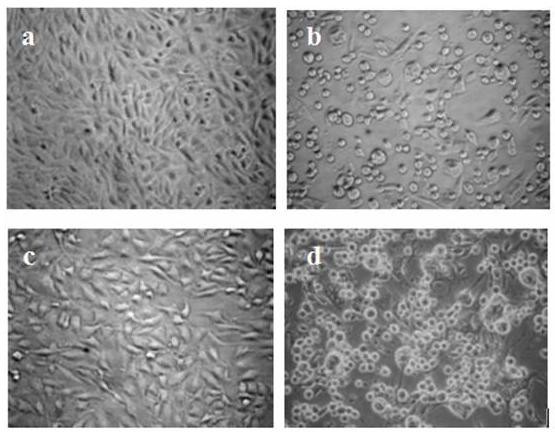
**Cell morphology and CPEs of 3A10-3F and BHK-21 cells under a phase-contrast microscope**. 3A10-3F cells (a) appeared morphologically similar to parental BHK-21 cells (c). After infected with HSV-1, 3A10-3F cells (b) appeared similar CPEs to normal BHK-21 cells infected with HSV-1(d) (Magnification: ×200).

In flow cytometry, the binding between 3A10-3F cells and JEV was 2.10% (Figure 4a), much lower than the binding between BHK-21 cells and JEV (48.84%, Figure 4b), clearly indicating that 3A10-3F cells are defective of JEV binding because of the compromise of normal surface protein functioning for JEV attachment and/or entry. Besides, 3A10-3F cells appear morphologically similar (Figure 5a) to normal BHK-21 cells (Figure 5c), and still susceptible to HSV-1 (SM64 strain) infection as they manifested the similar CPEs (Figure 5b) as rapidly as BHK-21 cells did (Figure 5d). The reason to choose HSV-1 is that HSV binds to its surface protein molecules definitely different from that of JEV on BHK-21 cells, thus excluding the possibility that several mosquito-borne flaviviruses (such as JEV, Dengue virus, West Nile virus, etc) may attach the same protein molecules when virus-host interaction occurs [16]. Therefore, the HSV-1 infection test reinforced that resistance of 3A10-3F cells to JEV infection was specific for JEV (machinery for gene expression appeared intact), and was much less probably resulted from the decreased virus replication within the cells but from less virus binding to cell surface molecules necessary for JEV attachment and/or entry.

All data above implied that (i) JEV was still permissive to replicate within 3A10-3F cells but at a low level; (ii) JEV might enter 3A10-3F cells through different molecule routes, possibly high affinity and low affinity receptors [10]; and (iii) 3A10-3F cells became resistant to JEV infection most likely due to the altered surface protein expression after chemical mutagenesis rather than the mutations of certain intracellular factors essential for JEV replication.

**Figure 6:**
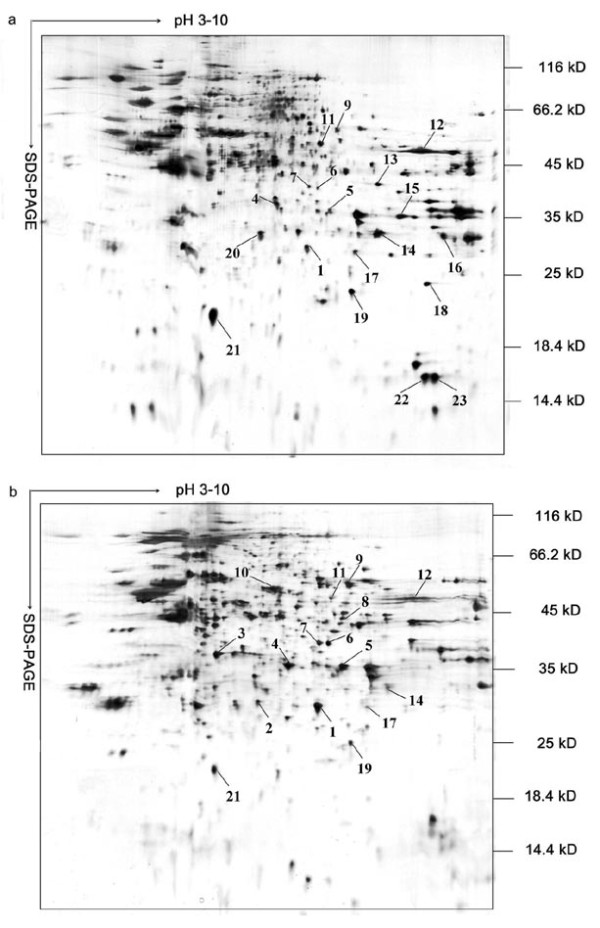
**Representative 2-DE maps of 3A10-3F and BHK-21 cell membrane protein extracts**. Membrane proteins from 3A10-3F (a) and BHK-21 cells (b) were separated by 2-DE and stained with sliver. 23 most differentially expressed protein spots were marked with arrows, and these spots were analyzed by LC-MS/MS. Three separate gels were prepared for each cell line.

Finally, two-dimensional electrophoresis (2-DE) coupled with mass spectrometry was used to determine those differentially expressed proteins between 3A10-3F and BHK-21 cell membrane protein extracts following Mirza's method [17]. Altogether 23 spots of differentially expressed proteins on SDS-PAGE gels were picked out (Figure 6) upon computerized sifting criteria (the smooth parameter was set to a value of 2; the minimum area was above 8 pixels; the saliency parameter was experimentally adjusted to 1), including six up-regulated spots (11, 12, 14, 17, 19, 21) and six down-regulated spots (1, 4-7, 9) in 3A10-3F cells compared with BHK-21 cells, seven spots (13, 15, 16, 18, 20, 22, 23) solely expressed in 3A10-3F cells and four spots (2, 3, 8, 10) in BHK-21 cells respectively. These spots were subject to mass spectrometry analysis in combination with a mouse peptide database (NCBInr) searching, and the results indicated that 14 spots among the 23 samples (spots 1, 3-5, 7, 10-12, 14-16, 20, 22, and 23) were determined with good peptide coverage and significant scoring (Additional file 1 table S1). Not surprisingly, not all proteins extracted were membrane proteins, but all proteins with most altered abundance must have been disclosed. With such certitude, four membrane proteins from 3A10-3F cells recognized by mass spectrometry, annexin 1 and annexin 2, voltage-dependent anion channel (VDACs) 1 and 2, were of our particular interests.

Annexins are a family of structurally related proteins whose common properties are to bind both phospholipids and cellular membranes in a calcium-dependent manner [18]. Annexin 1 involves in diverse cellular roles, including membrane fusion, exocytosis, differentiation, apoptosis, calcium channels, inflammatory reactions, and interaction with cytoskeletal proteins [19]. To date, few but one report [20] referred the role of annexin 1 in viral infection, stating that infectious pancreatic necrosis virus (IPNV) infection to fish cell increased the expression of salmon annexin 1, and the increased expression of salmon annexin 1 inhibited the apoptosis of IPNV-infected cells and supported the growth of IPNV in cells. In addition, annexin 2 had been identified as a receptor for cytomegalovirus (CMV) and respiratory syncytial virus (RSV) [21,22], and also promoted entry of HIV-1 into cells and proper assembly of HIV-1 in cells [23,24]. In our study, the expression of annexin 1 and annexin 2 were found to be significantly reduced on 3A10-3F cells, possibly suggesting that they may be involved in JEV attachment and/or entry.

VDACs is a multigene family of evolutionarily conserved and well characterized porins found in outer mitochondrial membranes of all eukaryotes [25], where they control homeostasis by transport of ATP and ADP [26]. Numerous research groups also reported the presence of VDAC proteins in the plasma membrane of various cell types [27,28]. VDAC 1 contributed to ATP transport across the plasma membrane of murine cells [29], and could act as NADH-ferricyanide reductase to inhibit the release of synthesized ATP and greatly decrease the activity of exogenous NADH/cytochrome-c system of intact mitochondria [30], however the significance of its total absence from 3A10-3F on JEV infection is not reported. Mammalian VDAC 2 exhibited other more biological activities, such as interaction with Bcl-2 family proteins, critical regulators of apoptosis [31]. In our study, VDAC 2 should not be a candidate for JEV binding molecule as it displayed an increased expression (16-fold) on 3A10-3F cells.

Nevertheless, some membrane molecules other than annexins and VDACs may not be excluded to mediate JEV entry into cells. It is not unusual that a virus particle utilizes multiple surface proteins during cell entry [32-34]. Several viruses utilize at least two different molecules to interact with their host cells via: (i) the binding receptors, merely serve as attachment factors that concentrate or recruit viruses on cell surface; (ii) co-receptors, that are used by the virus after binding to the cells, not only bind viruses but are also responsible for directing the bound viruses into endocytic pathways and for transmitting followed signals to the cytoplasm [35].

Taken together, the mutated cell line 3F10-3F from parental BHK-21 cells became less susceptible to JEV infection, and the discrepancy of membrane proteins between daughter and parental cells thus provides important clues to further investigate the individual role of the membrane proteins in JEV infection on BHK cells.

## List of abbreviations

BHK: baby hamster kidney; CPE: cytopathic effect; FITC: fluorescein isothiocyanate; HSV: herpes simplex virus; IFA: indirect immunofluorescence assay; ICR-191: (6-chloro-9-[3-(2-chloroethylamino)propylamino]-2-methoxyacridine; JEV: Japanese encephalitis virus; LC-MS: liquid chromatography-mass spectrometry; moi: multiplicity of infection; VDAC: voltage-dependent anion channels protein

## Competing interests

The authors declare that they have no competing interests.

## Authors' contributions

TD and JR conceived the study and drafted the manuscript. TD carried out the chemical mutation on BHK-21 cells. WZ did RT-PCR. JR carried out IFA, plaque formation assay, flow cytometry, and 2-DE. TD and JR did bioinformatic analysis. WM and JR proofread the manuscript. All authors read and approved the final manuscript.

## Supplementary Material

Additional file 1 **Table 1: Comparison of LC-MS/MS recognized proteins between 3A10-3F and BHK-21 cells**. A wider table describing recognized protein properties in MS Word .doc format. Click here for file
