# Supplementary material for: Identification of a mutated BHK-21 cell line that became less susceptible to Japanese encephalitis virus infection
Source: Virol J. 2011 Mar 14;8:115. doi: 10.1186/1743-422X-8-115 (PMC3064645; doi:10.1186/1743-422X-8-115)
Supplement: Additional file 1 — Table 1: Comparison of LC-MS/MS recognized proteins between 3A10-3F and BHK-21 cells. A wider table describing recognized protein properties in MS Word .doc format. [file 1743-422X-8-115-S1.DOC]

| Table 1. Comparison of LC-MS/MS recognized proteins between 3A10-3F and BHK-21 cells. | | | | | | | | | | |
| --- | --- | --- | --- | --- | --- | --- | --- | --- | --- | --- |
| *Spot*  *No* | *Accession*  *Number*  *(NCBI-Prot)* | *Protein names* | *Coverage(%)/ matched*  *peptides* | *Score* | *Theoretical*  *Mass (kD)* | *Theoretical*  *pI* | *Regulation*  *(BHK-21 as reference gel)* | *N-fold** | *Location* | *Function* |
| 1 | 114326546 | phosphoglycerate mutase 1 | 50.79 / 35 | 350 | 28.8 | 6.67 | Down | -5.3 | Cytoplasm | Metabolism |
| 3 | 6671569 | Acidic ribosomal phosphoprotein P0 | 23.03 / 9 | 90 | 34.2 | 5.91 |  | +100 | Cytoplasm | Ribosomal |
| 4 | 124517663 | annexin A1 | 11.85 / 12 | 120 | 38.7 | 6.97 | Down | -6.7 | Membrane | Calcium-binding |
| 5 | 6996913 | annexin A2 | 33.63 / 29 | 290 | 38.6 | 7.55 | Down | -11.3 | Membrane | Calcium-binding |
| 7 | 6754994 | poly(rC) binding protein 1 | 18.54 / 10 | 100 | 37.4 | 6.66 | Down | -2.4 | Cytoplasm | Signal transduction |
| 10 | 126521835 | chaperonin subunit 2 (β) | 31.4 / 36 | 360 | 57.4 | 5.97 |  | +100 | Cytoplasm | Protein binding |
| 11 | 6680027 | glutamate dehydrogenase 1 | 26.70 / 28 | 280 | 61.3 | 8.05 | Up | +3 | Cytoplasm | Metabolism |
| 12 | 6680748 | ATP synthase, H+ transporting, mitochondrial F1 complex, α subunit | 29.84 / 47 | 470 | 59.7 | 9.22 | Up | +2.6 | Cytoplasm | Metabolism |
| 14 | 6755965 | voltage-dependent anion channel 2 (VDAC 2) | 20.68 / 17 | 170 | 31.7 | 7.44 | Up | +16 | Membrane | ion transportation |
| 15 | 1351911 | ALDR_MOUSE Aldose reductase (AR) | 23.73 / 7 | 70 | 35.7 | 6.71 |  | -100 | Cytoplasm | Metabolism |
| 16 | 10720404 | Voltage-dependent anion channel 1 (VDAC 1) | 14.19 / 4 | 40 | 32.3 | 8.55 |  | -100 | Membrane | ion transportation |
| 20 | 31982169 | 3-hydroxybutyrate dehydrogenase, type 1 | 16.62 / 9 | 90 | 38.2 | 9.14 |  | -100 | Cytoplasm | Metabolism |
| 22 | 6679439 | peptidylprolyl isomerase A | 39.02 / 8 | 80 | 17.9 | 7.73 |  | -100 | Cytoplasm | Metabolism |
| 23 | 6679439 | peptidylprolyl isomerase A | 30.49 / 9 | 90 | 17.9 | 7.73 |  | -100 | Cytoplasm | Metabolism |
| *+100 represents spot only present on BHK-21 reference gel. -100 represents spot only present on 3A10-3F sample gel. | | | | | | | | | | |
